# Supplementary material for: Motivation matters: How enrollment motives shape doctoral experiences and career aspirations
Source: PLoS One. 2025 Sep 23;20(9):e0330679. doi: 10.1371/journal.pone.0330679 (PMC12456774; doi:10.1371/journal.pone.0330679)
Supplement: S2 Data — (DOCX) [file pone.0330679.s002.docx]

**S2. Appendix 1**

**Table A1. Goodness-of-fit statistics for latent class analysis models**

| No. of classes | AIC | BIC | $G^{2}$ | $\chi^{2}$ |
| --- | --- | --- | --- | --- |
| 1 | 12785.46 | 13027.25 | 845.46 | 3232.45 |
| 2 | 13005.79 | 13124.12 | 1113.79 | 7218.40 |
| 3 | 12848.60 | 13028.65 | 932.59 | 3450.42 |
| 4 | 12785.46 | 13027.25 | 845.46 | 3232.59 |
| 5 | 12770.29 | 13073.81 | 806.28 | 4270.61 |


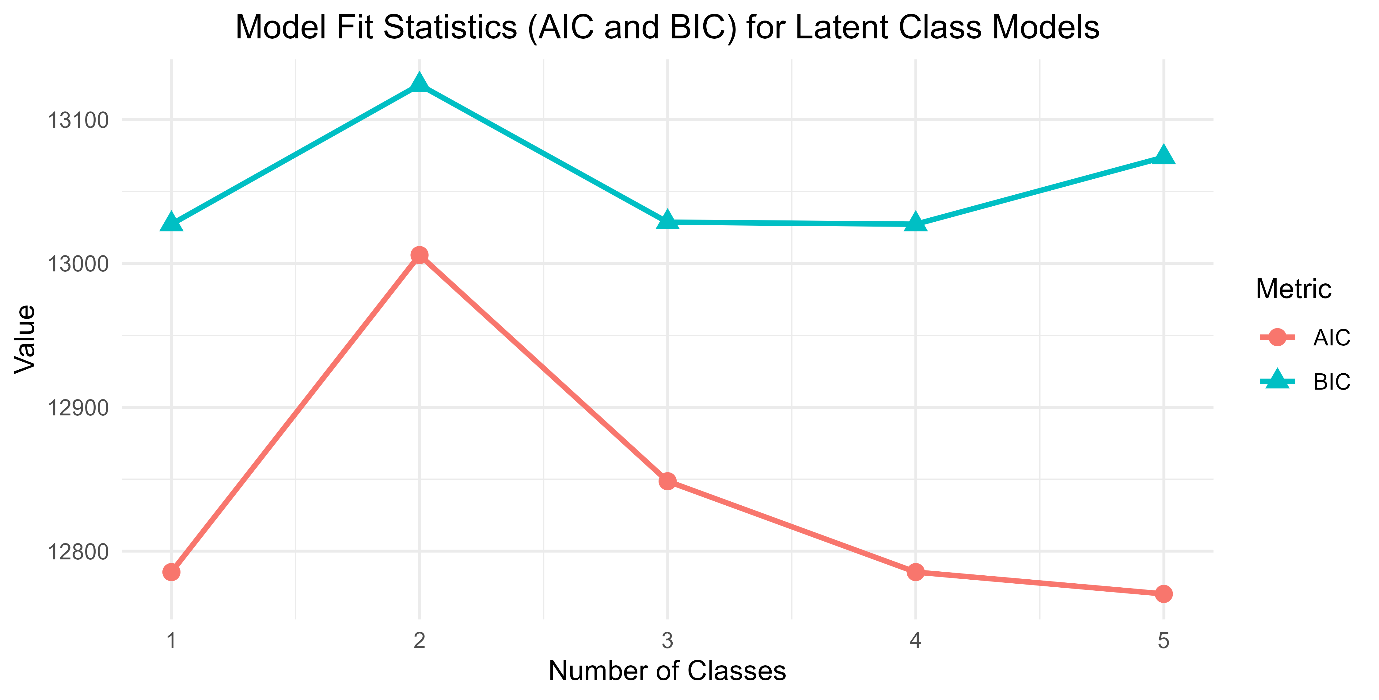


**Fig A1. Model fit indices (AIC and BIC) for latent class models with 1 to 5 classes.**

The four-class solution demonstrates a joint minimum of both AIC and BIC. Starting from the five-class model, BIC begins to increase, indicating a decline in model parsimony. Based on this fit pattern, as well as considerations of interpretability, we selected the four-class model for further analysis.

**Table A2. Average posterior probabilities by assigned latent class**

| Assigned Class | Mean P(Class 1) | Mean P(Class 2) | Mean P(Class 3) | Mean P(Class 4) |
| --- | --- | --- | --- | --- |
| 1 | 0.767 | 0.002 | 0.181 | 0.049 |
| 2 | 0.001 | 0.861 | 0.128 | 0.009 |
| 3 | 0.048 | 0.075 | 0.790 | 0.086 |
| 4 | 0.029 | 0.065 | 0.201 | 0.705 |

Each row represents the average posterior probabilities of class membership for respondents assigned to a particular class. High diagonal values and low off-diagonal values indicate strong class separation and good model classification quality.
