# Supplementary material for: Motivation matters: How enrollment motives shape doctoral experiences and career aspirations
Source: PLoS One. 2025 Sep 23;20(9):e0330679. doi: 10.1371/journal.pone.0330679 (PMC12456774; doi:10.1371/journal.pone.0330679)
Supplement: Attachment — Submitted filename: Russian doc students.docx [file pone.0330679.s004.docx]

First sentence is not related to the topic

Please open with what your argument – the second sentence can be reframed that those who enter doctoral programs are intrinsically motivated

Please say academic motivation and non-academic motivation ----as compared to intrinsic motivation and extrinsic motivation

Honestly I would simply start at line 147 then explain the academic vs non-academic in that section

The new first page is an extremely long paragraph—it honestly have several separate ideas that need to become their own paragraphs

This lit review is extremely short—only 1 ½ pages. That is not enough information to support this study

For non-Russians we will not know what this mean-- “5-100” or 206 “Priority-2030”

Please either eliminate or expand

LCA is used to pull apart classes—why it is LCA. Here is an AI summary

Latent Class Analysis (LCA) provides a statistical framework for uncovering hidden subgroups within a population based on patterns of observed data. It's a mixture model that assumes an underlying, unobserved categorical variable divides individuals into distinct, mutually exclusive latent classes. The rationale behind LCA is **to identify these latent classes and understand how they relate to observed behaviors or characteristics**.

So yes it has a categorical variable but the goal is to find latent classes and why their motivations are here in terms of getting a doctoral degree

Figure 2 is not what I see in other papers

Can you look and see if you can find a clearer way to diagram the LCA?

You have never explained self determination theory—it was mentioned once in the lit and now once in results—this needs to be set up in the lit review

Your lack of confidence variable should be defined in the method section

Line 329—again you need a well defined Data Analytic section that sets up all of these analyses

They keep popping up and were not set up in the Data Analytic section in the method

You do not detail the catPCA’s that you discuss. We need to see everything that you have done – this data should not be in the appendix but the early method section

Logistic regression—it would be helpful to say what this regression shows---in terms of academic vs non-academic motivation

Why didn’t you do a multiple logistic regression rather than all of these separate ones

However, a more detailed breakdown of our results reveals several concerning issues. 419 First, there is evidence of a Matthew effect: leading universities attract the most motivated 420 doctoral students, thereby exacerbating gaps and educational inequality. *What evidence are you using to support this?*

Second, employment in non-academic sectors is more frequently associated with an 425 amotivated profile, and the practice of combining work and study is widespread in Russia [63]. 426 This presents a substantial risk: doctoral students may be unable to fully immerse themselves 427 in the university environment, making it more difficult to develop intrinsic motivation to 428 enhance persistence and achievement [54, 62]. This challenge, driven by the necessity of 429 balancing work and study, reignites the discussion about the insufficient funding of Russian 430 doctoral education [23, 68].

Amotivated should be non-acadmic I think

I am not sure that this is only a Russian issue. The online doctoral degrees that accept people without matching undergraduate/masters degrees is the same in the US. For example, a coach or a dancer getting an EdD in Education Leadership is the same. So I think online education is similar to your findings.

However, a more detailed breakdown of our results reveals several concerning issues. 419 First, there is evidence of a Matthew effect: leading universities attract the most motivated 420 doctoral students, thereby exacerbating gaps and educational inequality. –*is this because those universities offer assistantships to support students? That is happening in many places. Then you get part time students because they do not have tuition waivers or graduate assistantships*
