# Supplementary material for: Motivation matters: How enrollment motives shape doctoral experiences and career aspirations
Source: PLoS One. 2025 Sep 23;20(9):e0330679. doi: 10.1371/journal.pone.0330679 (PMC12456774; doi:10.1371/journal.pone.0330679)
Supplement: Attachment — Submitted filename: Response to reviewers.docx [file pone.0330679.s005.docx]

**Response to Editor and Reviewers**

Dear Editor, Dr. Clark,

Dear Reviewers,

We sincerely appreciate your deep engagement with the manuscript and your insightful comments. We hope that we managed to revise the text significantly in line with your suggestions. Below, we respond to them one by one and the changes made in the text.

**Academic Editor**

**1.** First sentence is not related to the topic. Please open with what your argument – the second sentence can be reframed that those who enter doctoral programs are intrinsically motivated

**Authors**: Thank you for the suggestion. In response, we removed the general opening sentence and revised the paragraph to begin with a more focused statement about students’ intrinsic motivation when entering doctoral programs.

**2.** Please say academic motivation and non-academic motivation — as compared to intrinsic motivation and extrinsic motivation

**Authors**: Thank you for the comment. As suggested, we replaced the terms academic/non-academic *motives* with academic/non-academic *motivation* to ensure clarity and conceptual consistency.

**3.** Honestly I would simply start at line 147 then explain the academic vs non-academic in that section

The new first page is an extremely long paragraph — it honestly have several separate ideas that need to become their own paragraphs. This lit review is extremely short—only 1 ½ pages. That is not enough information to support this study

**Authors:** Thank you for your feedback. In response, we restructured the literature review section by breaking down the original long paragraph into several shorter ones to improve clarity and focus. We also expanded the literature review by providing a more detailed discussion of academic and non-academic motivation in these paragraphs.

**4.** For non-Russians we will not know what this mean — “5-100” or 206 “Priority-2030”. Please either eliminate or expand.

**Authors**: Thank you for your comment. To make the text clearer for international readers, we have removed the reference to national program names and revised the sentence as follows: “*Additionally, 43% of the sample studied at leading universities, including federal universities, national research universities, and institutions participating in excellence programs*”.

**5.** LCA is used to pull apart classes — why it is LCA. Here is an AI summary:

“Latent Class Analysis (LCA) provides a statistical framework for uncovering hidden subgroups within a population based on patterns of observed data. It's a mixture model that assumes an underlying, unobserved categorical variable divides individuals into distinct, mutually exclusive latent classes. The rationale behind LCA is to identify these latent classes and understand how they relate to observed behaviors or characteristics.”

So yes it has a categorical variable but the goal is to find latent classes and why their motivations are here in terms of getting a doctoral degree.

**Authors**: Thank you for the suggestion. We have clarified the rationale for using Latent Class Analysis (LCA) in the revised ‘Data Analysis’ section. The new paragraph reads:

*To construct motivational profiles of doctoral students, we applied latent class analysis (LCA). LCA is a statistical technique that identifies an unobserved categorical variable based on patterns in observed categorical indicators. In our case, it provides a class (motivational profile), based on the presence or absence of various enrollment motivations. In a sense, it resembles factor analysis, but is specifically designed for categorical rather than continuous variables. This method enables the identification of coherent motivational profiles, which is more appropriate than analyzing motivations separately and aligns with previous studies [49, 59, 60]. Based on goodness-of-fit statistics and interpretability, we selected a four-class model (see Appendix 1 for details and visualisation). The labels for all classes were chosen by the authors based on item-response probabilities. The probability of selecting each motive for each class is illustrated in Figure 2.*

We hope that this clarification better communicates the analytical logic of our method. This person-centered approach allows us to capture the complexity of students’ motivational structures by identifying patterns that may not be evident through variable-centered methods.

**6.** Figure 2 is not what I see in other papers. Can you look and see if you can find a clearer way to diagram the LCA?

**Authors**: Thank you for your feedback. We reviewed relevant literature and consulted colleagues from the Computer Science department regarding best practices for visualizing LCA results. Bar charts illustrating conditional probabilities are widely used and considered effective for displaying latent class structures. For example:

1. Dong, Z., Wu, G., Liu, H., Chen, S., Bi, B., Zhang, F., ... & Tan, Y. (2025). Cardiovascular comorbidities in Chinese inpatients with schizophrenia spectrum disorders. *Schizophrenia, 11*(1), 22. <https://doi.org/10.1038/s41537-025-00576-3>
2. Sotres-Alvarez, D., Herring, A. H., & Siega-Riz, A. M. (2010). Latent class analysis is useful to classify pregnant women into dietary patterns. *The Journal of nutrition, 140*(12), 2253-2259. <https://doi.org/10.3945/jn.110.124909>
3. Zhou, W., He, MM., Wang, F. et al. (2023) Latent class analysis-derived classification improves the cancer-specific death stratification of molecular subtyping in colorectal cancer. *npj Precision Oncology, 7,* 60. <https://doi.org/10.1038/s41698-023-00412-w>
4. Zhao, X. (2023). Displaying Latent Classes in Figures: Consideration of Practices. *The Quantitative Methods for Psychology, 19*(2), 165-172. <https://doi.org/10.20982/tqmp.19.2.p165>

In other cases, authors rely solely on tabular presentation of conditional probabilities, such as:

Zhuchkova, S., Terentev, E., Saniyazova, A., & Bekova, S. (2023). Departmental academic support for doctoral students in Russia: Categorisation and effects. *Higher Education Quarterly, 77*(2), 215-231. <https://doi.org/10.1111/hequ.12389>

We chose a bar chart format because it enhances readability and makes class-specific motivational patterns more accessible to a broad readership. That said, if the editor has a particular style or format in mind (e.g., a profile plot or heatmap), we would be happy to adjust the figure accordingly.

**7.** You have never explained self determination theory—it was mentioned once in the lit and now once in results—this needs to be set up in the lit review.

**Authors:** Thank you for pointing this out. To address the issue, we added a dedicated Conceptual Framework section before the literature review, where we introduce self-determination theory in detail and explain how it informs the distinction between academic and non-academic motivation used in the study.

**8.** Your lack of confidence variable should be defined in the method section

**Authors**: Thank you for pointing this out. We have now moved the description of the “lack of confidence” variable to the appropriate section and clarified how it was constructed. The revised text reads as follows:

*The second model examines motivational profiles in relation to doctoral students’ self-assessed likelihood of successfully defending their dissertation. Following the approach proposed by Zhuchkova et al. [67], we constructed a variable called “lack of confidence” using categorical principal component analysis (CatPCA), which allows for the extraction of a continuous component from categorical indicators. The questionnaire items used, along with component loadings and communalities, are presented in Table 2.*

*TABLE 2*

*These items demonstrate a high level of consistency (Cronbach’s alpha = 0.89). The resulting index represents doctoral students’ unconfidence in successfully completing their program and defending their dissertation, with higher values indicating lower confidence.*

**9.** Line 329 — again you need a well defined Data Analytic section that sets up all of these analyses. They keep popping up and were not set up in the Data Analytic section in the method.

**Authors**: Thank you for this important observation. We have restructured the entire empirical section to make our analytical strategy clearer and more transparent to the reader. Instead of a separate subsection labeled “Data Analysis,” we now present a structured and sequential layout under a unified empirical section. This structure mirrors the logic of our analysis and allows readers to clearly follow each step:

- Sample and data collection – detailing the sampling procedure and data collection process.
- Motivational profiles of doctoral students – focused on the LCA: including the rationale, questionnaire items, model fit criteria, and description of the four identified classes.
- Portrait of groups – descriptive comparative analysis of how the motivational classes differ across socio-demographic and educational characteristics using chi-square tests.
- Motives, trajectories, and doctoral experience – regression analyses divided into four subparts, each with its own dependent variable, model specification, and interpretation:
  - Motivations and satisfaction – binary logistic regression examining class differences in satisfaction.
  - Motivations and self-assessed confidence – linear regression, including the construction of the “lack of confidence” index via CatPCA.
  - Motivations and career aspirations – binary logistic regression analyzing links between motivational classes and plans to pursue a research career.
  - Can experience reshape motivation? – regression on a subsample of students with “Unconscious motives,” identifying which components of doctoral experience contribute to research-oriented career aspirations.

We hope that this restructured section now provides a clearer and more systematic account of our data analytic approach.

**12**. You do not detail the catPCA’s that you discuss. We need to see everything that you have done – this data should not be in the appendix but the early method section

**Authors**: Thank you for the suggestion. We have now moved all relevant details about the CatPCA procedure from the appendix to the main Methods section to ensure transparency and reproducibility. The revised text now fully describes the construction of the six components related to the doctoral experience, including the procedure, rationale for model selection, and use of component scores in subsequent regression analyses. The corresponding factor loadings and communalities are now presented in Table X.

*To measure different aspects of doctoral experience, we used the following single-choice question: “To what extent are the following aspects present during your doctoral studies?” (Scale: 3 – “To a great extent”, 2 – “To some extent”, 1 – “Not at all present”). The question included 19 statements. For subsequent analysis, these statements were grouped into several components using categorical principal component analysis (catPCA). The analysis was conducted on a polychoric correlation matrix using the psych package in R. The promax rotation was applied, as the components of the doctoral experience were expected to be correlated.*

*To determine the optimal number of components, we compared several different models. A six-component model was ultimately selected due to its interpretability. It explains 78% of the variance in the original variables related to the doctoral experience. The variable “The need to use English or another foreign language” was excluded due to interpretation challenges. The resulting components capture distinct aspects of the doctoral experience:*

*1.	Interaction with supervisor;*

*2.	Research activities;*

*3.	International activities;*

*4.	Interaction with peers;*

*5.	Interaction with department;*

*6.	Educational workload.*

*Component loadings and communalities are presented in Table 5.*

*TABLE 5*

*Six variables were extracted – standardized factor scores of components, where higher values of these variables indicate a greater prominence of the corresponding aspects in students’ experiences. We use these six variables to find out which aspects of doctoral experience are related to research-oriented career aspirations among those who initially enrolled with non-academic motives.*

**12**. Logistic regression—it would be helpful to say what this regression shows---in terms of academic vs non-academic motivation.

**Authors**: Thank you for the suggestion. In our models, we do not rely on a binary academic vs. non-academic distinction. Instead, we use the four motivational profiles identified through latent class analysis as categorical predictors. This approach allows us to go beyond a simplistic dichotomy and capture more nuanced motivational patterns — including “Unconscious Motives” and “Everything Everywhere All at Once” profiles — and assess how each of them relates to doctoral satisfaction, confidence, and career aspirations.

By including a rich set of control variables in each model, we are able to estimate the independent contribution of each motivational profile while accounting for socio-demographic and institutional characteristics. We hope this makes the interpretation of the logistic regressions more meaningful and aligned with the complexity of doctoral motivation.

**13.** Why didn’t you do a multiple logistic regression rather than all of these separate ones?

**Authors**: Thank you for this question. We chose not to estimate a single multiple logistic regression because our models address distinct dependent variables, each capturing a different aspect of doctoral outcomes: satisfaction with studies, confidence in dissertation defense, and plans to pursue a research career. Statistically, these are conceptually and operationally separate constructs, and combining them into one model would not be appropriate.

Moreover, from a substantive perspective, analyzing these outcomes separately allows us to identify unique associations between motivational profiles and each specific aspect of the doctoral experience. For example, students with “Everything Everywhere All at Once” profiles are significantly more likely to plan a research career but do not differ in satisfaction or confidence, whereas “Unconscious Motives” consistently predict lower satisfaction, confidence, and career aspirations. Different sets of control variables also show significance across models, which enriches the interpretation and highlights how various factors matter differently depending on the outcome in question.

For these two reasons, separate models provide a clearer and more nuanced understanding of how motivation shapes doctoral trajectories across multiple dimensions. Our approach aligns with prior studies that examine distinct dimensions of doctoral outcomes separately (e.g., Sverdlik et al., 2018 <https://doi.org/10.28945/4113>, or Litalien & Guay, 2015 <https://doi.org/10.1016/j.cedpsych.2015.03.004>), recognizing that these aspects may be influenced by different mechanisms

**14. “**However, a more detailed breakdown of our results reveals several concerning issues. First, there is evidence of a Matthew effect: leading universities attract the most motivated doctoral students, thereby exacerbating gaps and educational inequality”. What evidence are you using to support this?

**Authors**: Thank you for this important question. The statement regarding a Matthew effect is based on the analysis presented in the ‘Portrait of groups’ section, where we examine the relationship between motivational profiles and university type using chi-squared tests. As shown in Figure 3, students from leading universities are significantly less likely to belong to the “Unconscious Motives” class — a profile characterized by unclear or non-academic goals, such as deferring military service. This suggests that students with stronger academic motivation are more likely to enter highly selective institutions, reinforcing institutional stratification in doctoral education.

A section in ‘Discussion’ now read as follows:

*However, a more detailed breakdown of our results reveals several concerning issues. First, there is evidence of a Matthew effect: leading universities attract the most motivated doctoral students, thereby exacerbating gaps and educational inequality. This is supported by our analysis in the “Portrait of groups” section (see Figure 3), which shows that students from leading universities are significantly less likely to belong to the “Unconscious Motives” class — a profile associated with unclear or non-academic enrollment goals, such as deferring military service. The overconcentration of doctoral education in leading universities in Russia [37], combined with unclear motives of doctoral students in non-elite universities, presents a significant challenge for regional universities in terms of (re)producing human capital.*

**15.** “Second, employment in non-academic sectors is more frequently associated with an amotivated profile, and the practice of combining work and study is widespread in Russia [63]. This presents a substantial risk: doctoral students may be unable to fully immerse themselves in the university environment, making it more difficult to develop intrinsic motivation to enhance persistence and achievement [54, 62]. This challenge, driven by the necessity of balancing work and study, reignites the discussion about the insufficient funding of Russian doctoral education [23, 68]”. Amotivated should be non-academic I think.

**Authors**: Thank you for this comment! We rephrased the sentence to be more coherent with latent classes labels: *“Second, employment in non-academic sectors is more frequently associated with unconscious motives, and the practice of combining work and study is widespread in Russia [63]”.*

**16.** I am not sure that this is only a Russian issue. The online doctoral degrees that accept people without matching undergraduate/masters degrees is the same in the US. For example, a coach or a dancer getting an EdD in Education Leadership is the same. So I think online education is similar to your findings.

**Authors**: We fully agree that the patterns we observe may resonate with broader international trends — including the rise of online and professional doctoral programs that attract students with diverse educational and career backgrounds. However, our study is focused specifically on the Russian doctoral education system, and we do not have sufficient empirical or conceptual grounding in online or professional doctorate models to make strong claims in that area.

That said, we have incorporated a brief acknowledgment of this possible parallel in the Discussion section, while being careful not to overextend our argument beyond the scope of our data and expertise. We hope this strikes the right balance between situating our findings in a wider conversation and maintaining analytical precision:

*While our findings are grounded in the context of Russian doctoral education, similar patterns may be observed in other systems that have experienced an expansion of non-traditional or professionally oriented doctoral pathways. For example, online EdD programs in countries like the United States often enroll candidates with diverse academic and career backgrounds [68], whose initial motivations may not align with traditional research-oriented goals. Although we do not explore these models in depth, the parallels suggest that the challenges associated with heterogeneous motivations are not unique to Russia and may warrant further comparative research.*

**17.** “However, a more detailed breakdown of our results reveals several concerning issues. First, there is evidence of a Matthew effect: leading universities attract the most motivated doctoral students, thereby exacerbating gaps and educational inequality”. Is this because those universities offer assistantships to support students? That is happening in many places. Then you get part time students because they do not have tuition waivers or graduate assistantships

**Authors**: Thank you for raising this point. While we agree that the availability of assistantships or tuition waivers can shape doctoral student enrollment patterns in many systems, our dataset does not contain direct information on institutional financial support mechanisms. Therefore, we are cautious not to draw causal conclusions about why more motivated students tend to enroll in leading universities.

However, existing research on the Russian context (e.g., [37] by Zhuchkova & Bekova) suggests that more selective universities benefit from greater institutional capacity, visibility, and academic reputation, which makes them more attractive to highly motivated applicants. As we note in the “Discussion” section, this leads to an overconcentration of doctoral education in elite institutions, while non-elite universities face challenges in recruiting students with clear academic goals — thereby contributing to structural inequality within the doctoral education system.
