# Supplementary material for: Motivation matters: How enrollment motives shape doctoral experiences and career aspirations
Source: PLoS One. 2025 Sep 23;20(9):e0330679. doi: 10.1371/journal.pone.0330679 (PMC12456774; doi:10.1371/journal.pone.0330679)
Supplement: Attachment — Submitted filename: Response_to_reviewers_auresp_3.docx [file pone.0330679.s006.docx]

**Response to Editor and Reviewers**

Dear Editor, Dr. Clark,

Dear Reviewers,

We sincerely appreciate your deep engagement with the manuscript and your comments. We hope that we managed to revise the text in line with your suggestions. Below, we respond to them one by one and highlight the changes made in the text.

**Reviewer #1:**

**1.** Lines 46, 169, 177, 267, 276, 285, and 294 - Add a space after the comma.

**Authors**: We added a space under the comma in the according lines.

**2.** Line 229 - Spell out what HSE stands for before introducing the acronym.

**Authors**: We spelled out that HSE stands for Higher School of Economics in line 229: The study received approval from the Institutional Review Board at the Higher School of Economics (HSE University). The authors accessed the data on November 28, 2024.

**3.** Lines 356, 375, 391, and 429 - The percentages under LB CI and UB CI should be written as 97.5%, not 97,5%.

**Authors:** We have corrected lines 356, 375, 391, and 429, replacing commas with dots.

**4.** Line 357 - In the Notes, the p value should be formatted as:

*p < 0.05; **p < 0.01; ***p < 0.001

instead of:

*p < 0,05; **p < 0,01; ***p < 0,001

**Authors**: We made the according correction, replacing commas with dots.

**5.** Line 376 - In the Notes, the p value should be written as:

p-value < 0.001

instead of:

p-value < 0,001

**Authors**: We made the correction, replacing commas with dots.
